# Association Between Adjuvant Chemotherapy Duration and Survival Among Patients With Stage II and III Colon Cancer: A Systematic Review and Meta-analysis

**DOI:** 10.1001/jamanetworkopen.2019.4154

**Published:** 2019-05-17

**Authors:** Devon J. Boyne, Colleen A. Cuthbert, Dylan E. O’Sullivan, Tolulope T. Sajobi, Robert J. Hilsden, Christine M. Friedenreich, Winson Y. Cheung, Darren R. Brenner

**Affiliations:** 1 Department of Community Health Sciences, Cumming School of Medicine, University of Calgary, Calgary, Alberta, Canada; 2 Department of Cancer Epidemiology and Prevention Research, CancerControl Alberta, Alberta Health Services, Calgary, Alberta, Canada; 3 Department of Oncology, Cumming School of Medicine, University of Calgary, Calgary, Alberta, Canada; 4 Department of Public Health Sciences, School of Medicine, Queen’s University, Kingston, Ontario, Canada; 5 Department of Medicine, Cumming School of Medicine, University of Calgary, Calgary, Alberta, Canada

## Abstract

**Importance:**

The results from the recent International Duration Evaluation of Adjuvant Therapy (IDEA) collaboration have led some clinicians to adopt shorter durations of adjuvant chemotherapy for patients with stage III colon cancer. The extent to which these findings are supported by other data is unknown.

**Objective:**

To conduct a systematic review and meta-analysis of randomized and observational studies investigating the association between the duration of adjuvant chemotherapy and survival among individuals diagnosed as having stage II and III colon cancer (PROSPERO protocol CRD42018108711]).

**Data Sources:**

Abstracts published in English between 2003 and 2018 within the MEDLINE, Embase, CENTRAL, and CINAHL databases were reviewed by 2 authors. Also searched were conference proceedings and the indexes of high-impact oncology journals.

**Study Selection:**

Studies were excluded if they did not present original data; focused on animal populations, on cancers in sites other than the colon, or on patients with stage 0, I, or IV disease; did not examine a 5-flourouracil–based monotherapy or combination therapy; or did not evaluate the association between treatment duration and survival. The search identified 2341 articles, from which 2 randomized trials and 20 observational studies were included in the meta-analysis.

**Data Extraction and Synthesis:**

This study followed the PRISMA and MOOSE reporting guidelines. The risk of bias was assessed by 2 authors using the Cochrane and Risk of Bias in Nonrandomized Studies of Interventions (ROBINS-I) tools. The results were synthesized using a random-effects model.

**Main Outcomes and Measures:**

The primary and secondary outcomes were overall survival and disease-free survival, respectively. It was hypothesized a priori that 3 months of chemotherapy would be as effective as 6 months of chemotherapy.

**Results:**

Twenty-two studies were included in the meta-analysis, representing 43 671 patients. The inclusion of patients with stage II disease or with rectal cancer was identified as a source of heterogeneity. After restricting the analysis to patients with stage III colon cancer, there was no association between the duration of chemotherapy and overall survival among studies involving FOLFOX (leucovorin calcium [folinic acid], fluorouracil, and oxaliplatin) or CAPOX (capecitabine plus oxaliplatin) regimens (hazard ratio [HR], 0.80; 95% CI, 0.58-1.09). Among studies focused exclusively on monotherapy, the standard 6-month regimen relative to a 3-month regimen was associated with improved survival (HR, 0.59; 95% CI, 0.52-0.68).

**Conclusions and Relevance:**

Shortened durations of chemotherapy may reduce survival among patients with stage III colon cancer prescribed monotherapy but not a combination regimen.

## Key Points

**Question:**

Are shortened durations of adjuvant chemotherapy associated with decreased survival among patients with stage II and III colon cancer?

**Findings:**

In this systematic review and meta-analysis of 22 studies comprising 43 671 patients, shortened durations of adjuvant chemotherapy were not associated with worse survival in studies involving combination regimens (the current standard of care) among patients with stage III colon cancer. Conversely, the standard 6 months of chemotherapy was associated with improved survival among patients prescribed monotherapy.

**Meaning:**

Shorter durations of adjuvant chemotherapy may not adversely alter survival among patients with stage III colon cancer treated with combination therapy; patients prescribed monotherapy should be encouraged to complete the entire 6 months of treatment.

## Teaser

This systematic review and meta-analysis investigates the association between the duration of adjuvant chemotherapy and survival among individuals diagnosed as having stage II and III colon cancer.

## Introduction

Identifying shorter durations of chemotherapy that minimize adverse effects while maintaining clinical efficacy could lead to improved patient outcomes and cost savings. To address this issue, a recent pooled analysis of 6 randomized trials sought to assess the noninferiority of 3 months of adjuvant FOLFOX (leucovorin calcium [folinic acid], fluorouracil, and oxaliplatin) or CAPOX (capecitabine plus oxaliplatin) chemotherapy relative to the 6-month standard among patients with stage III colon cancer.^1^ Although the results from this collaboration were inconclusive in the entire study population, noninferiority was observed among certain subgroups, namely, patients with “low risk” stage III disease (T1-3 and N1) and patients treated with CAPOX.^1^ These findings have resulted in clinical practice guidelines recommending shorter durations of adjuvant chemotherapy within this patient population.^2^ It is important to note that the International Duration Evaluation of Adjuvant Therapy (IDEA) collaboration^1^ examined disease-free survival and was conducted within a clinical trial setting, which may have limited generalizability. In addition, the results from this investigation are not generalizable to adjuvant monotherapies, which are still prescribed to patients unable to tolerate oxaliplatin. As such, the results from studies examining adjuvant monotherapies or overall survival and from investigations conducted within real-world settings may help to support the findings from the IDEA collaboration^1^ and strengthen recommendations within clinical practice guidelines.

Existing review articles on this topic are limited. A meta-analysis^3^ published in 2010 found that 9 to 12 months of adjuvant chemotherapy was not more effective than 3 to 6 months. That review article did not compare durations of chemotherapy that were less than 6 months, include patients treated with FOLFOX or CAPOX (the current standards of care among patients with stage III colon cancer),^2^ or synthesize the results from observational studies. A more recent review article^4^ examining adjuvant chemotherapy duration was conducted; however, that review article was not systematic and did not include a meta-analysis of the evidence base. To address these limitations, we performed a systematic review and meta-analysis of randomized and observational studies that have examined the association between the duration of adjuvant chemotherapy and overall or disease-free survival among patients with stage II and III colon cancer. We hypothesized a priori that 3 months of chemotherapy would be as effective as 6 months of chemotherapy.

## Methods

We previously registered a protocol for this systematic review and meta-analysis with PROSPERO (CRD42018108711), the international prospective register of systematic reviews.^5^ The present study followed the Preferred Reporting Items for Systematic Reviews and Meta-analyses (PRISMA)^6^ and the Meta-analysis of Observational Studies in Epidemiology (MOOSE)^7^ guidelines.

### Eligibility Criteria

To be eligible for inclusion, studies had to report the findings from a randomized clinical trial or observational study investigating the association between the duration of a fluorouracil (including prodrugs, such as capecitabine or tegafur) monotherapy or combination therapy (ie, CAPOX or FOLFOX) and overall or disease-free survival among individuals diagnosed as having stage II or III colon cancer. Studies were excluded if they were a review article, commentary, editorial, protocol, case series, or case report; were conducted in a nonhuman (eg, mice) or laboratory (eg, cell line) population; were focused exclusively on patients with cancers in sites other than the colon; were focused on early-stage (stage 0 or I) or metastatic (stage IV) tumors; failed to examine adjuvant chemotherapy duration or discontinuation; examined a therapy other than those previously described; or if a survival outcome was not reported.

### Information Sources and Search Strategy

We systematically searched the MEDLINE, Embase, CENTRAL, and CINAHL databases using a query that was developed by one of us (D.J.B.) in collaboration with a research librarian. This query was previously registered with PROSPERO and is available in eAppendix 1 in the Supplement. The search was conducted on August 10, 2018, and was restricted to abstracts published in English between 2003 and 2018.

In addition to our database search, we scanned the references of the included articles, the conference proceedings of the American Society of Clinical Oncology, and the indexes of the following oncology journals: *Annals of Oncology*, *JAMA Oncology*, *Journal of Clinical Oncology*, *Cancer*, *Journal of Oncology Practice*, and *Lancet Oncology*.

### Study Selection

The assessment of eligibility was conducted independently by 2 of us (D.J.B. and C.A.C.). Titles and abstracts were first screened for relevance, and then a full-text screen was undertaken. At both stages, agreement between the 2 reviewers was assessed using the percentage agreement and κ statistics. Disagreements between the 2 were resolved through discussion. Non–English language articles with abstracts written in English that were deemed eligible for full-text review were translated and assessed for eligibility.

### Data Collection Process

Data extraction was carried out by the first author (D.J.B.). The results of this extraction were reviewed by another author (D.E.O.) for accuracy. We attempted to contact authors in cases where the hazard ratio (HR) and corresponding 95% CI were not reported or readily estimable using the available data. These emails were sent on December 6, 2018. Articles were not included in the quantitative synthesis if the authors were unable to provide an estimate suitable for meta-analysis or if the authors could not be reached.

### Study Quality

The risk of bias was assessed independently by 2 of us (D.J.B. and D.E.O.) using the Cochrane Collaboration’s tool for assessing the risk of bias in randomized trials^8^ and the Risk of Bias in Nonrandomized Studies of Interventions (ROBINS-I) tool.^9^ The final decision regarding the risk of bias for each study was reached through discussion by the 2 authors. Regarding the ROBINS-I tool, we developed a list of confounders a priori that would be relevant to the studies included in this systematic review and meta-analysis. We separated confounders into the following 2 categories according to our certainty that their adjustment was necessary to produce an unbiased effect estimate: (1) important confounders—type of chemotherapy, age, sex, site (colon or rectum), and tumor stage; and (2) potential confounders—comorbidity, performance status, body mass index, tumor location, tumor grade, period of diagnosis, time from surgery to the initiation of chemotherapy, presence of postoperative complications, dose delays, and dose reductions. We did not identify any relevant cointerventions. With respect to bias arising from selection into the study, a low-risk designation was given if the index time was defined as the start of chemotherapy initiation and if the inclusion of participants was not potentially related to the exposure and outcome.

### Statistical Analysis

To account for differences in the definitions of chemotherapy completion, we converted each measure of association to an estimate of the change in the log HR per 3 additional months of chemotherapy. We first estimated the median number of months of chemotherapy for each duration category using the middle value of the range of the category. In situations where the number of cycles was used to define categories of duration, clinical standards were used to determine the corresponding duration (ie, 1 cycle of FOLFOX given every 2 weeks, 1 cycle of capecitabine monotherapy or CAPOX given every 3 weeks, or 1 cycle of 5-fluorouracil monotherapy given every 4 weeks). As done in a previous systematic review and meta-analysis,^10^ we estimated the change in the log HR per 3 additional months of chemotherapy as follows: β = [ln(HR) / (*x*_1_ − *x*_0_)]*3, where *x*_1_ and *x*_0_ refer to the median duration (in months) of the upper and lower categories, respectively. We similarly estimated the corresponding SE as follows: SE(β) = {(ln[upper 95% CI] – ln[lower 95% CI]) / ([*x*_1_ − *x*_0_]*3.92)}*3. When estimating the β coefficient and its SE, the multiplication by 3 was done so that they corresponded to the change in the log HR per 3 additional months of chemotherapy as opposed to the change in the log HR per 1 additional month of chemotherapy. Additional information regarding the derivation of effect estimates in situations where the HR and 95% CI were not directly reported can be found in eAppendix 2 in the Supplement.

The primary and secondary outcomes of interest were overall survival and disease-free survival, respectively. To facilitate the pooling of the results from randomized and observational studies, we focused on per-protocol effect estimates. Given the expected between-study heterogeneity due to differences in patient populations, a random-effects DerSimonian-Laird model was used to estimate the average measure of association. In situations in which a study reported effect estimates for independent subgroups, the subgroups were treated as individual studies in the meta-analysis. In addition to 95% CIs, we also estimated the 95% prediction intervals. As previously discussed, prediction intervals should be reported when conducting random-effects meta-analysis because they provide a range of effect estimates that one would expect to observe in a clinical setting similar to those in the meta-analysis.^11^ The presence of publication bias was assessed via funnel plots and with the Begg test^12^ and the Egger test.^13^ We used the *I*^2^ statistic, the τ^2^ estimate, and a χ^2^ test to assess heterogeneity within this evidence base.^14^ To identify sources of heterogeneity, we conducted subgroup analyses by treatment-related variables (chemotherapy regimen and the middle period of diagnosis), patient characteristics (mean age and percentage female), tumor features (percentage with colon cancer and percentage with stage III cancer), and study characteristics (country, median follow-up, and risk of bias). Analyses were performed using the *metafor* package in R Studio (version 1.1.419).^15^ Statistical significance was defined at the .05 threshold, and all statistical tests were 2-sided.

## Results

A PRISMA flow diagram of the inclusion of studies is shown in eFigure 1 in the Supplement. Our database search identified 2341 publications after removal of duplicates. After an initial screen, 205 articles were reviewed in full, from which 34 studies (9 randomized trials^1,16,17,18,19,20,21,22,23^ and 25 observational studies^24,25,26,27,28,29,30,31,32,33,34,35,36,37,38,39,40,41,42,43,44,45,46,47,48^) were deemed eligible for inclusion. There was strong agreement between the 2 reviewers at both the initial screen and the full-text screen (percentage agreement >90% and κ > 0.6).

Among the 9 randomized trials, only 3 investigated durations of chemotherapy shorter than the current 6-month standard, specifically the IDEA collaboration^1^ and the studies by Chau et al^17^ and by Ito et al.^20^ Among these studies, per-protocol estimates were only available for the IDEA collaboration^1^ and for the study by Ito et al.^20^ The investigation by Chau et al^17^ did not examine different durations of the same treatment modality. Therefore, we were only able to include the results from the IDEA collaboration^1^ and from the study by Ito et al^20^ in the meta-analysis.

Among the 25 observational studies identified by our search strategy, 5 were excluded from the meta-analysis. Specifically, we excluded the studies by Ahn et al^25^ and by Yaich et al^47^ because we were unable to obtain an effect estimate suitable for inclusion in the meta-analysis and we were unable to reach these authors for further details regarding their findings. In addition, we excluded the studies by Satkunam et al^40^ and by Tashiro et al^43^ due to clinical heterogeneity in the exposure definition. Specifically, these researchers did not strictly examine the duration of the prescribed chemotherapy regimen; rather, they studied a composite exposure that included dose reductions and dose modifications, such as a periodic dose omission. Last, we excluded the study by Dobie et al^28^ because they explored cancer-specific mortality and did not report an estimate specific to overall survival or disease-free survival.

In total, 2 randomized trials and 20 observational studies were included in the meta-analysis,^1,20,24,26,27,29,30,31,32,33,34,35,36,37,38,39,41,42,44,45,46,48^ representing 43 671 patients diagnosed between 1987 and 2015. Of these 22 studies, 19 examined overall survival, and 12 examined disease-free survival. Descriptive characteristics are listed in Table 1. The complete data extracted from the individual studies are summarized in eTable 1 in the Supplement.

**Table 1.** Characteristics of 22 Studies Included in the Meta-analysis

Source	Country	Patients, No.	Period of Diagnosis	Follow-up, Median, y	Age, y^a^	No. (%)			Includes Patients Treated With FOLFOX or CAPOX
						Female	Colon Cancer^b^	Stage III	
IDEA collaboration,^1^ 2018	Multiple	10 395^c^	2007-2015	DFS, 3.5	Median (range), 64 (18-88)^d^	4532 (43.6)^d^	10 395 (100)	10 395 (100)	Yes
Ito et al,^20^ 2000	Japan	144	1987-1990	NR	NR	NR	88 (61.1)	NR	No
Ji et al,^32^ 2018	South Korea	147	2006-2014	NR	Mean (SD), 62 (10.1)	66 (44.9)	147 (100)	119 (81.0)	Yes
Laurent et al,^36^ 2018	France	153	2009-2013	OS, 2.4	Median (IQR), 66 (56-72)	67 (43.8)	134 (87.6)	111 (72.5)	Yes
Cespedes Feliciano et al,^26^ 2017	United States	533	2006-2011	NR	Mean (SD), 59 (11.3)	291 (54.6)	533 (100)	458 (85.9)	Yes
Hwang et al,^31^ 2017^e^	South Korea	24 874^d^	2011-2014	NR	NR	NR	NR (100)	NR (<100)	Yes
van Erning et al,^46^ 2017	the Netherlands	352	2005-2012	OS, 5.4; DFS, 2.9	Median (range), 74 (70-NR)	169 (48.0)	352 (100)	352 (100)	Yes
Tsai et al,^45^ 2016	Taiwan	213	2005-2012	OS, 4.5	Median (range), 62 (29-88)	99 (46.5)	213 (100)	213 (100)	Yes
Hassan et al,^30^ 2015	Malaysia	86	2004-2009	OS, 7.0	Mean (SD), 59 (14.9)	41 (47.7)	86 (100)	58 (67.4)	Yes
Kumar et al,^35^ 2015	Canada	616	2006-2010	OS, 4.2	Median (range), 62 (26-80)	295 (47.9)	616 (100)	616 (100)	Yes
Sgouros et al,^41^ 2015	Greece	508	1995-2011	OS, 5.3	Median (range), 66 (29-87)	229 (45.1)	370 (72.8)	294 (57.9)	Yes
Sun et al,^42^ 2016	Canada	217	2008-2012	NR	67	91 (41.9)	158 (72.8)	118 (54.4)	No
Kim et al,^33^ 2014	Canada	268	2004-2010	OS, 3.3	Median (range), 73 (65-NR)	136 (50.7)	268 (100)	268 (100)	Yes
Tsai et al,^44^ 2013	Taiwan	716	1996-2001	OS, 6.0	Median (range), 58 (21-86)	349 (48.7)	352 (49.2)	716 (100)	No
Figer et al,^29^ 2011	Israel	398	1990-1995	OS, 8.3	Median (range), 63 (24-76)	188 (47.2)	322 (80.9)	201 (50.5)	No
Ahmed et al,^24^ 2010	Canada	663	1993-2000	OS, 4.6	Median (range), 66 (25-86)	276 (41.6)	364 (54.9)	540 (81.4)	No
Yun et al,^48^ 2010	Korea	173	2005-2007	DFS, 3.3	Median (range), 58 (29-78)	59 (34.1)	173 (100)	135 (78.0)	No
Chapuis et al,^27^ 2009	Australia	104	1992-2004	OS, 6.4	Median, 66	NR	104 (100)	104 (100)	No
Qiu et al,^39^ 2009	China	216	2003-2007	DFS, 3.0	Median, 52	82 (38.0)	130 (60.2)	144 (66.7)	Yes
Kornmann et al,^34^ 2008^f^	Germany	855	1992-1999	OS, 4.6	Median, 62	447 (52.3)	855 (100)	787 (92.0)	No
Morris et al,^37^ 2007	Australia	461	1994-2001	OS, 3.0	Median, 68	223 (48.4)	461 (100)	461 (100)	No
Neugut et al,^38^ 2006	United States	1579	1995-1999	NR	Median (range), 74 (65-NR)^d^	900 (57.0)^d^	1579 (100)	1579 (100)	No

Abbreviations: CAPOX, capecitabine plus oxaliplatin; DFS, disease-free survival; FOLFOX, leucovorin calcium (folinic acid), fluorouracil, and oxaliplatin; IDEA, International Duration Evaluation of Adjuvant Therapy; IQR, interquartile range; NR, not reported; OS, overall survival.

^a^ The mean was used in situations in which the median was unavailable.

^b^ The other types of cancer were rectal cancers.

^c^ Refers to the per-protocol population.

^d^ Estimated using available information about the underlying study population.

^e^ The results from this study were published in a conference abstract, and a full-text article was not available at the time of publication. The sample size refers to both patients with colon cancer and patients with rectal cancer, which is an overestimate of the number of patients with colon cancer included in their analysis.

^f^ These investigations were observational studies nested within existing randomized trials.

The results from the study quality assessment are listed in Table 2, and the additional details regarding the reasons for the risk of bias assignment within individual domains are listed in eTable 2 in the Supplement. For the 2 randomized trials included in this systematic review and meta-analysis, we had some concerns regarding the risk of bias in the per-protocol effect estimate due to nonadherence because potential confounding arising from between-arm differences in adherence (there were higher levels of adherence in the shorter-duration arms) was not addressed using appropriate statistical methods.^49^ The risk of bias in the other domains was deemed to be low. Among the 20 observational studies included in the meta-analysis, 12 were judged to have a serious risk of bias due to confounding (n = 9) or selection into the study (n = 3). The remaining 8 studies were deemed to have a moderate risk of bias. None of the studies in this evidence base were judged to have a high or critical risk of bias.

**Table 2.** Risk of Bias Assessment of 22 Studies Included in the Meta-analysis^a^

Source	Bias Arising From the Randomization Process or Due to Confounding^b^	Bias Due to Selection Into the Study	Bias in Classification of Interventions	Bias Due to Deviations From the Intended Intervention	Bias Due to Missing Outcome Data^c^	Bias in Measurement of Outcomes	Bias in Selection of the Reported Results	Overall Risk
IDEA collaboration,^1^ 2018	Low	NA	NA	Some concerns	Low	Low	Low	Some concerns
Ito et al,^20^ 2000	Low	NA	NA	Some concerns	Low	Low	Low	Some concerns
Ji et al,^32^ 2018	Serious	Low	Low	Low	Low	Low	Moderate	Serious
Laurent et al,^36^ 2018	No information (moderate)	Low	Low	Low	No information (low)	Low	Moderate	Moderate
Cespedes Feliciano et al,^26^ 2017	Serious	Moderate	Low	Low	No information (low)	Low	Moderate	Serious
Hwang et al,^31^ 2017	No information (moderate)	No information (low)	Low	Low	No information (low)	Low	Moderate	Moderate
van Erning et al,^46^ 2017	Moderate	Moderate	Low	Low	No information (low)	Low	Moderate	Moderate
Tsai et al,^45^ 2016	Moderate	Serious	Low	Low	Low	Low	Moderate	Serious
Hassan et al,^30^ 2015	Serious	Moderate	Low	Low	No information (low)	Low	Moderate	Serious
Kumar et al,^35^ 2015	Moderate	Low	Low	Low	No information (low)	Low	Moderate	Moderate
Sgouros et al,^41^ 2016	Serious	Low	Low	Low	Moderate^d^	Low	Moderate	Serious
Sun et al,^42^ 2015	Serious	Moderate	Low	Low	No information (low)	Low	Moderate	Serious
Kim et al,^33^ 2014	Serious	Low	Low	Low	No information (low)	Low	Moderate	Serious
Tsai et al,^44^ 2013	Moderate	Moderate	Low	Low	No information (low)	Low	Moderate	Moderate
Figer et al,^29^ 2011	Serious	Moderate	Low	Low	Low	Low	Moderate	Serious
Ahmed et al,^24^ 2010	Moderate	Serious	Low	Low	Low	Low	Moderate	Serious
Yun et al,^48^ 2010	Serious	Moderate	Low	Low	No information (low)	Low	Moderate	Serious
Chapuis et al,^27^ 2009	Serious	Moderate	Low	Low	Low	Low	Moderate	Serious
Qiu et al,^39^ 2009	Moderate	No information (moderate)^e^	Low	Low	No information (low)	Low	Moderate	Moderate
Kornmann et al,^34^ 2008	Moderate	Low	Low	Low	No information (low)	Low	Moderate	Moderate
Morris et al,^37^ 2007	No information (moderate)	Moderate	Low	Low	No information (low)	Low	Moderate	Moderate
Neugut et al,^38^ 2006	Moderate	Serious	Low	Low	No information (low)	Low	Moderate	Serious

Abbreviations: IDEA, International Duration Evaluation of Adjuvant Therapy; NA, not applicable.

^a^ For the 2 randomized trials included in this evidence base, the columns refer to the Cochrane Collaboration’s tool for assessing the risk of bias in randomized trials. For the remaining observational studies, the columns refer to the Risk of Bias in Nonrandomized Studies of Interventions (ROBINS-I) tool.

^b^ Risk of bias arising from the randomization process is presented for randomized clinical trials, and the risk of bias due to confounding is presented for observational studies. All observational studies with the “no information” designation adjusted for variables using regression but did not specify which variables they controlled for in the analysis. In each case, we assumed that the authors adjusted for the important confounders we identified a priori.

^c^ Studies with the “no information” designation did not provide the number of patients lost to follow-up or the amount of missing data. Given the reliance on medical records, administrative databases, and cancer registries, we thought that the risk of bias due to missingness would be low.

^d^ More than 10% of patients had missing data.

^e^ The index time used to define survival time was not described.

**Figure 1.**
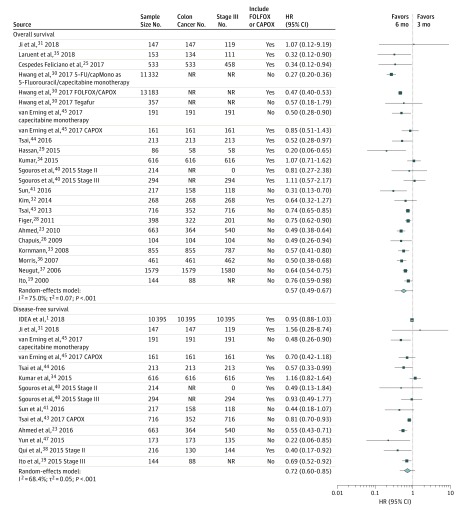
Meta-analysis of the Estimated Hazard of Death Among Patients With Stage II and III Colon Cancer Treated With 6 Months of Adjuvant Chemotherapy Relative to Those Who Received 3 Months of Adjuvant Chemotherapy The edges of the diamond refer to the 95% CI, and the error bars surrounding summary estimates refers to the 95% prediction interval. The size of the individual point estimates corresponds to the model weights. The prediction intervals are as follows: 0.33 to 1.00 for overall survival^20,24,26,27,29,30,31,32,33,34,35,36,37,38,41,42,44,45,46^ and 0.45 to 1.14 for disease-free survival.^1,20,24,32,35,39,41,42,44,45,46,48^ CAPOX indicates capecitabine plus oxaliplatin; FOLFOX, leucovorin calcium (folinic acid), fluorouracil, and oxaliplatin; HR, hazard ratio; and NR, not reported.

The results from the meta-analysis of overall survival and disease-free survival are summarized in a forest plot (Figure 1). For both outcomes, there was a suggestion that 6 months of adjuvant chemotherapy relative to 3 months was associated with improved survival; however, the 95% prediction intervals included the null: the HR for overall survival was 0.57 (95% CI, 0.49-0.67 and 95% prediction interval, 0.33-1.00), and the HR for disease-free survival was 0.72 (95% CI, 0.60-0.85 and 95% prediction interval, 0.45-1.14). There was no evidence of publication bias for overall survival according to a qualitative examination of the funnel plots (eFigure 2 in the Supplement) or the Begg test (Kendall τ = −0.14; *P* = .37) or the Egger test (*z* = −0.71; *P* = .48). With respect to disease-free survival, there was a suggestion of possible publication bias arising from a lack of small studies with estimates centered around the null (Kendall τ = −0.10; *P* = .67 for the Begg test and *z* = −2.06; *P* = .04 for the Egger test) (eFigure 3 in the Supplement).

There was moderate to substantial heterogeneity within this evidence base for the primary outcome (*I*^2^ = 75.0%; τ^2^ = 0.07; *P* < .001). The results from the subgroup analysis are summarized in Table 3. The inclusion of patients with stage II disease in these studies was a statistically significant source of heterogeneity. Specifically, the magnitude of the association was attenuated among studies focused exclusively on patients with stage III disease. Although not considered statistically significant sources of heterogeneity, subgroup analyses suggested that the period of diagnosis, mean age, inclusion of patients with rectal cancer, country of study, and median follow-up were also sources of heterogeneity. Specifically, the magnitude of the association appeared to be more pronounced in studies that were conducted (1) within more recent periods, (2) among older populations, (3) exclusively in patients with colon cancer, (4) in Asian populations, and (5) in those with less follow-up. The chemotherapy regimen, proportion of women, and risk of bias did not appear to be sources of heterogeneity according to these subgroup analyses.

**Table 3.** Subgroup Analysis of Studies Examining the Association Between Chemotherapy Duration and Overall Survival Among Patients With Stage II and III Colon Cancer

Variable	Estimates, No.^a^	Hazard Ratio (95% CI)	95% Prediction Interval	*I*^2^ Statistic, %	*P* Value of Ratio
**Treatment Variables**					
Chemotherapy regimen					
Included patients treated with combination therapy	11	0.62 (0.45-0.85)	0.28-1.36	64.0	.53
Patients treated with monotherapy only	12	0.55 (0.46-0.66)	0.31-0.97	80.2	
Middle period of diagnosis					
2001 Onward	15	0.53 (0.40-0.70)	0.23-1.20	70.7	.21
Before 2001	8	0.64 (0.56-0.72)	0.48-0.84	55.3	
**Patient Characteristics**					
Age, median, y					
≥65	11	0.57 (0.49-0.67)	0.42-0.88	29.0	.15
<65	8	0.69 (0.57-0.83)	0.48-0.99	47.9	
% Female					
≥50	4	0.62 (0.54-0.71)	0.54-0.71	0	.82
<50	14	0.63 (0.53-0.76)	0.39-1.02	59.1	
**Tumor Characteristics**					
% Colon cancer					
100	15	0.53 (0.44-0.65)	0.30-0.95	71.3	.10
<100	8	0.67 (0.55-0.80)	0.45-0.99	58.3	
% Stage III					
100	10	0.68 (0.58-0.79)	0.49-0.94	44.9	.01
<100	12	0.46 (0.36-0.59)	0.24-0.89	75.2	
**Study Characteristics**					
Country^b^					
Asian	8	0.50 (0.36-0.70)	0.23-1.12	87.8	.27
Non-Asian	15	0.62 (0.53-0.72)	0.42-0.91	48.5	
Median follow-up, y					
≥5	8	0.72 (0.61-0.84)	0.55-0.94	27.7	.14
<5	7	0.58 (0.46-0.73)	0.36-0.92	50.5	
ROBINS-I overall risk of bias^c^					
Moderate	10	0.55 (0.43-0.72)	0.26-1.15	85.5	.66
Serious	12	0.59 (0.49-0.71)	0.40-0.87	42.1	

Abbreviation: ROBINS-I, Risk of Bias in Nonrandomized Studies of Interventions.

^a^ There were a total of 23 estimates from 19 studies. Some estimates do not sum to 23 because some studies did not report the patient characteristic of interest and, as a result, were excluded from the analysis (see Table 1).

^b^ Asian countries include Japan, Malaysia, South Korea, and Taiwan. Non-Asian countries include Australia, Canada, France, Germany, Greece, Israel, the Netherlands, and the United States.

^c^ Conducted within observational studies only (excluded 1 randomized trial examining disease-free survival).

**Figure 2.**
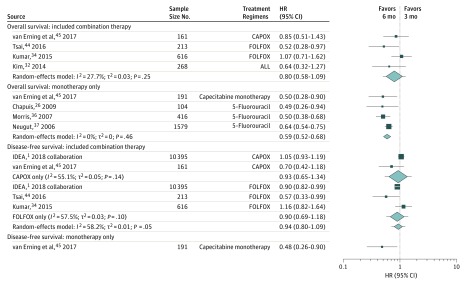
Meta-analysis of the Estimated Hazard of Death Among Patients With Stage III Colon Cancer Treated With 6 Months of Adjuvant Chemotherapy Relative to Those Who Received 3 Months of Adjuvant Chemotherapy The edges of the diamond refer to the 95% CI, and the error bars surrounding summary estimates refers to the 95% prediction interval. The size of the individual point estimates corresponds to the model weights. The prediction intervals are as follows: 0.50 to 1.27 for overall survival: included combination therapy,^33,35,45,46^ 0.52 to 0.68 (same as 95% CI because estimated τ^2^ = 0) for overall survival: monotherapy only,^27,37,38,46^ and 0.71 to 1.24 for disease-free survival: included combination therapy.^1,35,45,46^ The study by Kim et al^33^ included patients treated with FOLFOX, CAPOX, 5-fluorouracil, and capecitabine monotherapy. CAPOX indicates capecitabine plus oxaliplatin; FOLFOX, leucovorin calcium (folinic acid), fluorouracil, and oxaliplatin; and HR, hazard ratio.

Given the presence of heterogeneity and the identification of tumor stage and site as sources of heterogeneity, we repeated our primary analyses after restricting the analysis to study populations consisting exclusively of patients with stage III colon cancer. Because combination therapy is the current standard of care for patients with stage III colon cancer,^2^ we stratified these analyses by whether the investigation included patients treated with FOLFOX or CAPOX or was conducted exclusively among patients prescribed monotherapy (Figure 2). With respect to overall survival, there was little to no heterogeneity and no evidence of an association among studies involving combination regimens (HR, 0.80; 95% CI, 0.58-1.09; 95% prediction interval, 0.50-1.27). However, among studies of adjuvant monotherapy, 6 months of chemotherapy was associated with improved overall survival relative to 3 months (HR, 0.59; 95% CI, 0.52-0.68; 95% prediction interval, 0.52-0.68). With respect to disease-free survival, there remained some heterogeneity among studies of combination therapy, so we also further stratified by type of regimen. Only 1 study examined the association between monotherapy duration and disease-free results. In general, the results for disease-free survival were similar to those for overall survival.

## Discussion

Herein, we present the results from the first-ever systematic review and meta-analysis to date of randomized and nonrandomized studies examining durations of adjuvant chemotherapy shorter than 6 months and their association with survival among patients with stage II and III colon cancer. Given the large degree of heterogeneity, it would have been inappropriate to use a single estimate to characterize the results from the underlying evidence base. Because tumor stage and site were identified as sources of heterogeneity, we restricted our analyses to estimates specific to patients with stage III colon cancer and further stratified by chemotherapy regimen. The results from these subgroup analyses suggested that 3 months of chemotherapy relative to 6 months may not be associated with diminished survival among patients with stage III colon cancer prescribed adjuvant FOLFOX or CAPOX, but it may be associated with reduced survival among patients prescribed an adjuvant monotherapy. Estimates from observational studies examining the duration of FOLFOX (n = 2) or CAPOX (n = 1) and disease-free survival among patients with stage III colon cancer were imprecise but consistent with the subgroup analyses of the IDEA collaboration.^1^

These findings support recommendations made by the National Comprehensive Cancer Network^2^ of 3 to 6 months of adjuvant combination therapy, depending on the regimen and tumor substage, or 6 months of adjuvant monotherapy if the patient is not a suitable candidate for oxaliplatin therapy. When revising these guidelines to account for the recent findings from the IDEA collaboration,^1^ concerns were raised regarding the underestimation of the treatment effect arising from the use of disease-free survival as opposed to overall survival.^2^ Although disease-free survival has been shown to be a good surrogate end point for overall survival in this patient population,^50^ we found that the magnitude of the average measure of association tended to be larger when examining overall survival as opposed to disease-free survival, which provides some support for these concerns. This discrepancy may be larger if publication bias was present among the estimates of disease-free survival as suggested in our analyses. However, differences between studies with respect to the degree of bias and precision may also account for this discrepancy.

Additional research could further strengthen current clinical practice guidelines. Mature results from the IDEA collaboration^1^ focused on overall survival will help to address concerns regarding the reliance on disease-free survival. Ancillary per-protocol analyses of the IDEA collaboration^1^ that address nonadherence and censoring using modern statistical techniques, such as structural nested failure-time models and inverse probability of censoring weights, would also help to strengthen the evidence.^49^ Future observational studies from large real-world data sets could examine the association between chemotherapy duration and overall survival among strata defined within the subgroup analyses of the IDEA collaboration.^1^ Such granularity from well-conducted studies could further strengthen the evidence surrounding the targeting of adjuvant chemotherapies for patients with stage III colon cancer. Although adjuvant chemotherapy is offered to high-risk patients with stage II colon cancer,^51^ additional research regarding the optimal duration of adjuvant treatment within this patient population is needed.

### Recommendations for Future Research

Based on our review of the evidence to date, we propose a series of recommendations for improving the conduct of studies of adjuvant chemotherapy duration in real-world settings. Future studies could explore the duration of chemotherapy as an ordinal variable with 3 or more groups to allow for the exploration of a threshold effect. We had initially planned to conduct a nonlinear dose-response meta-analysis, but only 5 studies^34,37,38,39,45^ in the evidence base explored 3 or more categories of chemotherapy duration. To facilitate further meta-analysis, the median duration, the number of events, and the total number of patients or person-years at risk within each exposure category should be reported.^52^ In addition, studies should attempt to minimize the risk of bias due to confounding by adjusting for a greater number of confounders. The identification of sets of confounders that could be controlled for in the analysis can be guided by a directed acyclic graph.^53,54^ Researchers should be aware that comparisons of different durations of chemotherapy in real-world settings may be limited by immortal time bias.^55^ Specifically, there will be a period of immortal time among the longer-duration group and thus a survival benefit if the start of follow-up is defined as the date of diagnosis, the date of surgery, or the date of chemotherapy initiation and the exposure is treated as time invariant. To address this issue, we recommend that the index time be defined as the date of chemotherapy initiation and that the duration of treatment be modeled as a time-varying covariate. Among the observational studies included in this evidence base, only 1 study^36^ modeled chemotherapy duration as a time-varying covariate. Some studies excluded patients lost to follow-up, which may have resulted in selection bias. These patients should be included in the analyses, and inverse probability of censoring weights could be used to address differential loss to follow-up.^56^ In addition, the number of patients censored and lost to follow-up should be reported so that risk of bias can be better assessed. This information was not available in several of the studies included in this systematic review and meta-analysis. When comparing durations of chemotherapy, differences regarding dose reductions, omissions, or delays should be explored within the analysis. We propose that such differences may be conceptualized as different versions of the treatment or as time-varying confounders.^57^

### Limitations

This investigation has limitations that should be recognized. First, the results of this meta-analysis are likely biased due to residual confounding. A large proportion of the observational studies included in the meta-analysis (45%) were judged to have a serious risk of bias because they did not adjust for 1 or more of the important confounders we identified a priori. Second, the absence of an association among the subset of studies examining overall survival may be due to a lack of statistical power arising from a limited sample size. Third, the results of our meta-analysis may not be generalizable to patients with stage II disease. Although identified as a source of heterogeneity, there were insufficient data to examine the association of interest among patients with stage II colon cancer. As such, our results should only be generalized to patients with stage III colon cancer.

## Conclusions

While additional research is needed, the evidence to date suggests that shortened durations of adjuvant chemotherapy may not negatively alter survival among patients with stage III colon cancer who are prescribed FOLFOX or CAPOX. Conversely, the standard 6 months of adjuvant chemotherapy is associated with improved survival among patients treated who are prescribed monotherapy.

## Notes

Supplement. **eAppendix 1.** Search Strategy **eAppendix 2.** Additional Information Regarding the Derivation of Effect Estimates **eFigure 1.** PRISMA Flow Diagram **eFigure 2.** Funnel Plot Assessing Publication Bias Among Studies of Overall Survival **eFigure 3.** Funnel Plot Assessing Publication Bias Among Studies of Disease-Free Survival **eTable 1.** Raw Data Extracted From the Studies Included in the Meta-analysis (n = 21) **eTable 2.** Reason for Risk of Bias Assignment in the Confounding and Selection Into the Study Domains **eReferences.**
