# Supplementary material for: Association Between Adjuvant Chemotherapy Duration and Survival Among Patients With Stage II and III Colon Cancer: A Systematic Review and Meta-analysis
Source: JAMA Netw Open. 2019 May 17;2(5):e194154. doi: 10.1001/jamanetworkopen.2019.4154 (PMC6537824; doi:10.1001/jamanetworkopen.2019.4154)
Supplement: Supplement. — eAppendix 1. Search Strategy eAppendix 2. Additional Information Regarding the Derivation of Effect Estimates eFigure 1. PRISMA Flow Diagram eFigure 2. Funnel Plot Assessing Publication Bias Among Studies of Overall Survival eFigure 3. Funnel Plot Assessing Publication Bias Among Studies of Disease-Free Survival eTable 1. Raw Data Extracted From the Studies Included in the Meta-analysis (n = 21) eTable 2. Reason for Risk of Bias Assignment in the Confounding and Selection Into the Study Domains eReferences. [file jamanetwopen-2-e194154-s001.pdf]

Supplementary Online Content

Boyne DJ, Cuthbert CA, O'Sullivan DE, et al. Association between adjuvant chemotherapy duration and survival among patients with stage II and III colon cancer: a systematic review and meta-analysis. *JAMA Netw Open*. 2019;2(5):e194154.
doi:10.1001/jamanetworkopen.2019.4154

eAppendix 1. Search Strategy

eAppendix 2. Additional Information Regarding the Derivation of Effect Estimates

eFigure 1. PRISMA Flow Diagram

eFigure 2. Funnel Plot Assessing Publication Bias Among Studies of Overall Survival

eFigure 3. Funnel Plot Assessing Publication Bias Among Studies of Disease-Free Survival

eTable 1. Raw Data Extracted From the Studies Included in the Meta-analysis (n = 21)

eTable 2. Reason for Risk of Bias Assignment in the Confounding and Selection Into the Study Domains

eReferences.

This supplementary material has been provided by the authors to give readers additional information about their work.

eAppendix 1. Search Strategy

Database(s): **Ovid MEDLINE(R) and Epub Ahead of Print, In-Process & Other Non-Indexed Citations and Daily** 1946 to August 09, 2018
Search Strategy:

#	Searches
1	(Colo* adj2 (cancer or tumour* or tumor* or neoplasm* or carcinoma* or adenocarcinoma*)).tw,kf.
2	exp Colorectal Neoplasms/
3	((Adjuvant or postoperative or post-operative or postsurgical or post-surgical or "after surger*") adj2 (therap* or treatment* or chemotherap*)).tw,kf.
4	FOLFOX.tw,kf.
5	XELOX.tw,kf.
6	CAPOX.tw,kf.
7	5-FU.tw,kf.
8	Oxaliplatin.tw,kf.
9	Fluorouracil.tw,kf.
10	Capecitabine.tw,kf.
11	Leucovorin.tw,kf.
12	exp Chemotherapy, Adjuvant/
13	Duration.tw,kf.
14	Discontinu*.tw,kf.
15	(Cycles adj3 (completed or received or omission or receipt or enough or number*)).tw,kf.
16	Withdrawal.tw,kf.
17	Complian*.tw,kf.
18	Adherence.tw,kf.
19	Terminat*.tw,kf.
20	((Completed or completion or complete) adj3 (chemotherapy or therapy or treatment)).tw,kf.
21	1 or 2
22	3 or 4 or 5 or 6 or 7 or 8 or 9 or 10 or 11 or 12
23	13 or 14 or 15 or 16 or 17 or 18 or 19 or 20
24	21 and 22 and 23
25	limit 24 to (english language and yr="2003 -Current")

© 2019 Boyne DJ et al. *JAMA Network Open*.

Database(s): **Embase** 1974 to 2018 August 09
Search Strategy:

#	Searches
1	(Colo* adj2 (cancer or tumour* or tumor* or neoplasm* or carcinoma* or adenocarcinoma*)).tw,kw.
2	exp colorectal cancer/
3	((Adjuvant or postoperative or post-operative or postsurgical or post-surgical or "after surger*") adj2 (therap* or treatment* or chemotherap*)).tw,kw.
4	FOLFOX.tw,kw.
5	XELOX.tw,kw.
6	XELOX.tw,kw.
7	CAPOX.tw,kw.
8	5-FU.tw,kw.
9	Oxaliplatin.tw,kw.
10	Fluorouracil.tw,kw.
11	Capecitabine.tw,kw.
12	Leucovorin.tw,kw.
13	exp adjuvant chemotherapy/
14	Duration.tw,kw.
15	Discontinu*.tw,kw.
16	(Cycles adj3 (completed or received or omission or receipt or enough or number*)).tw,kw.
17	Withdrawal.tw,kw.
18	Complian*.tw,kw.
19	Adherence.tw,kw.
20	Terminat*.tw,kw.
21	((Completed or completion or complete) adj3 (chemotherapy or therapy or treatment)).tw,kw.
22	exp treatment duration/
23	1 or 2
24	3 or 4 or 5 or 6 or 7 or 8 or 9 or 10 or 11 or 12 or 13
25	14 or 15 or 16 or 17 or 18 or 19 or 20 or 21 or 22
26	23 and 24 and 25
27	limit 26 to (english language and yr="2003 -Current")
28	limit 27 to (conference abstract or conference paper or "conference review")
29	27 not 28

© 2019 Boyne DJ et al. *JAMA Network Open*.

Database(s): **EBM Reviews - Cochrane Central Register of Controlled Trials** July 2018
Search Strategy:

#	Searches
1	(Colo* adj2 (cancer or tumour* or tumor* or neoplasm* or carcinoma* or adenocarcinoma*)).tw,kf.
2	exp Colorectal Neoplasms/
3	((Adjuvant or postoperative or post-operative or postsurgical or post-surgical or "after surger*") adj2 (therap* or treatment* or chemotherap*)).tw,kf.
4	FOLFOX.tw,kf.
5	XELOX.tw,kf.
6	CAPOX.tw,kf.
7	5-FU.tw,kf.
8	Oxaliplatin.tw,kf.
9	Fluorouracil.tw,kf.
10	Capecitabine.tw,kf.
11	Leucovorin.tw,kf.
12	exp Chemotherapy, Adjuvant/
13	Duration.tw,kf.
14	Discontinu*.tw,kf.
15	(Cycles adj3 (completed or received or omission or receipt or enough or number*)).tw,kf.
16	Withdrawal.tw,kf.
17	Complian*.tw,kf.
18	Adherence.tw,kf.
19	Terminat*.tw,kf.
20	((Completed or completion or complete) adj3 (chemotherapy or therapy or treatment)).tw,kf.
21	1 or 2
22	3 or 4 or 5 or 6 or 7 or 8 or 9 or 10 or 11 or 12
23	13 or 14 or 15 or 16 or 17 or 18 or 19 or 20
24	21 and 22 and 23
25	limit 24 to (english language and yr="2003 -Current")

© 2019 Boyne DJ et al. *JAMA Network Open*.

Database(s): **CINAHL**
Search Strategy:

#	Searches
1	TI ((Colo* W2 (cancer or tumour* or tumor* or neoplasm* or carcinoma* or adenocarcinoma*))) OR AB ((Colo* W2 (cancer or tumour* or tumor* or neoplasm* or carcinoma* or adenocarcinoma*)))
2	(MH "Colorectal Neoplasms+")
3	TI (((Adjuvant or postoperative or post-operative or postsurgical or post-surgical or "after surger*") W2 (therap* or treatment* or chemotherap*))) OR AB (((Adjuvant or postoperative or post-operative or postsurgical or post-surgical or "after surger*") W2 (therap* or treatment* or chemotherap*)))
4	TI FOLFOX OR AB FOLFOX
5	TI XELOX OR AB XELOX
6	TI CAPOX OR AB CAPOX
7	TI 5-FU OR AB 5-FU
8	TI Oxaliplatin OR AB Oxaliplatin
9	TI Fluorouracil OR AB Fluorouracil
10	TI Capecitabine OR AB Capecitabine
11	TI Leucovorin OR AB Leucovorin
12	(MH "Chemotherapy, Adjuvant+")
13	TI Duration OR AB Duration
14	TI Discontinu* OR AB Discontinu*
15	TI ((Cycles W3 (completed or received or omission or receipt or enough or number*))) OR AB ((Cycles W3 (completed or received or omission or receipt or enough or number*)))
16	TI Withdrawal OR AB Withdrawal
17	TI Complian* OR AB Complian*
18	TI Adherence OR AB Adherence
19	TI Terminat* OR AB Terminat*
20	TI (((Completed or completion or complete) W3 (chemotherapy or therapy or treatment))) OR AB (((Completed or completion or complete) W3 (chemotherapy or therapy or treatment)))
21	(MH "Treatment Duration+")
22	1 or 2
23	3 or 4 or 5 or 6 or 7 or 8 or 9 or 10 or 11 or 12
24	13 or 14 or 15 or 16 or 17 or 18 or 19 or 20 or 21
25	22 and 23 and 24 (**Limiters** - Published Date: 20030101-20181231)

© 2019 Boyne DJ et al. *JAMA Network Open*.

eAppendix 2. Additional Information Regarding the Derivation of Effect Estimates

In situations where the hazard ratio and corresponding variance estimates were unavailable, we derived an estimate using the number of individuals within each exposure group, the total number of events, and the p-value from the log-rank test using the method described by Tierney et al. (2007).[1] If the authors provided effect estimates comparing different chemotherapy durations relative to a referent group not of interest to this publication (i.e. individuals who received no chemotherapy), we derived an effect estimate using the following formula: $HR_{A\ vs.\ B} = HR_{A\ vs.\ C} / HR_{B\ vs.\ C}$. We used the following formula to estimate the variance of the foregoing effect estimate: $var(ln(HR_{A\ vs.\ B}) = var(ln(HR_{A\ vs.\ c}) – ln(HR_{B\ vs.\ C})) = var(ln(HR_{A\ vs.\ C}) + var(ln(HR_{B\ vs.\ C})) – 2*cov(var(ln(HR_{A\ vs.\ C})), var(ln(HR_{B\ vs.\ C}))$. When applying this formula, we assumed a covariance of zero since the covariance was not directly estimable using the reported data. Assuming a positive covariance, this approach will bias the variance estimate in the conservative direction by inflating the variance and reducing the precision in the effect estimate.

The former method was used for one study (Moris et al. (2007))[2] and the method described by Tierney et al. (2007) was used for six studies.[3-8]

© 2019 Boyne DJ et al. *JAMA Network Open*.

eFigure 1. PRISMA Flow Diagram

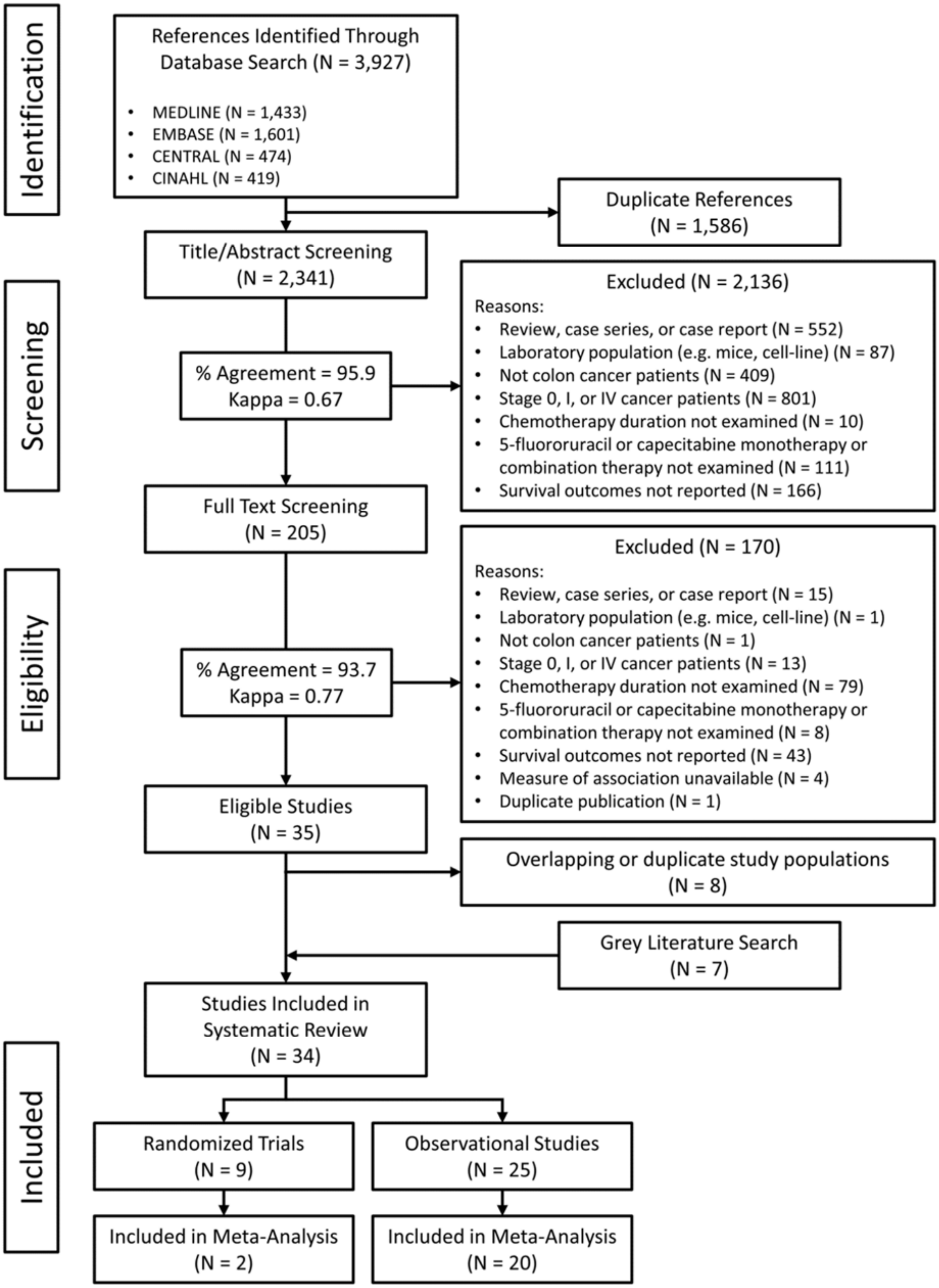

© 2019 Boyne DJ et al. *JAMA Network Open*.

eFigure 2. Funnel Plot Assessing Publication Bias Among Studies of Overall Survival

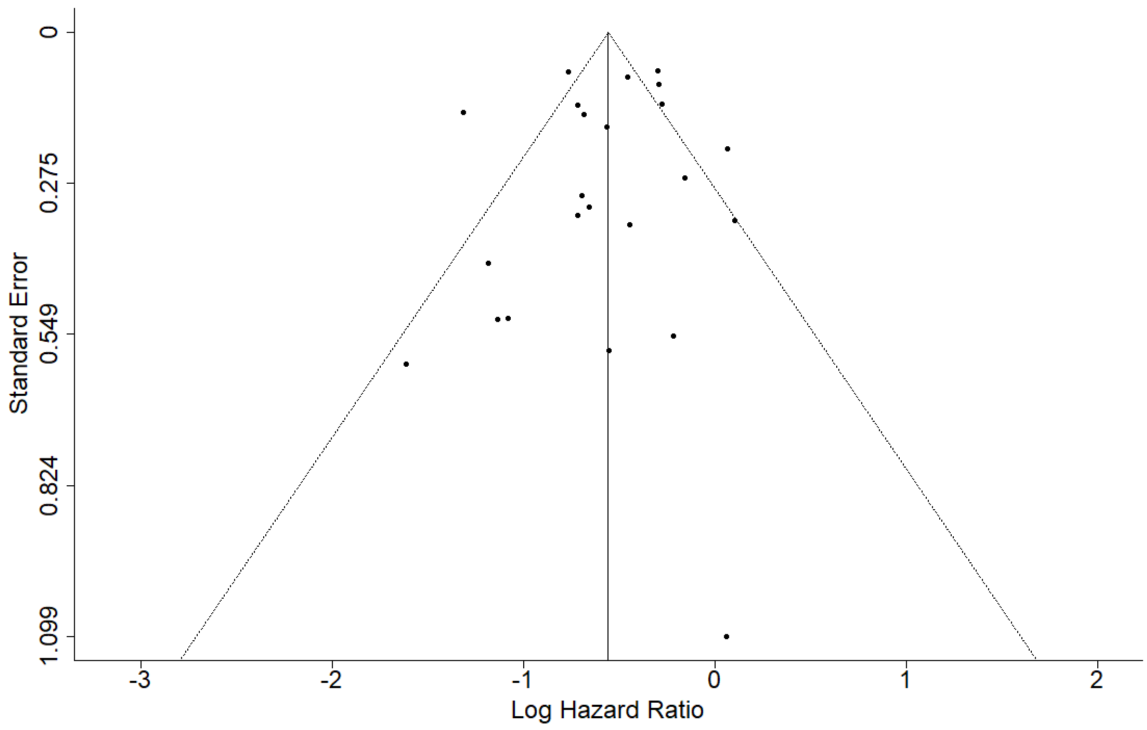

© 2019 Boyne DJ et al. *JAMA Network Open*.

eFigure 3. Funnel Plot Assessing Publication Bias Among Studies of Disease-Free Survival

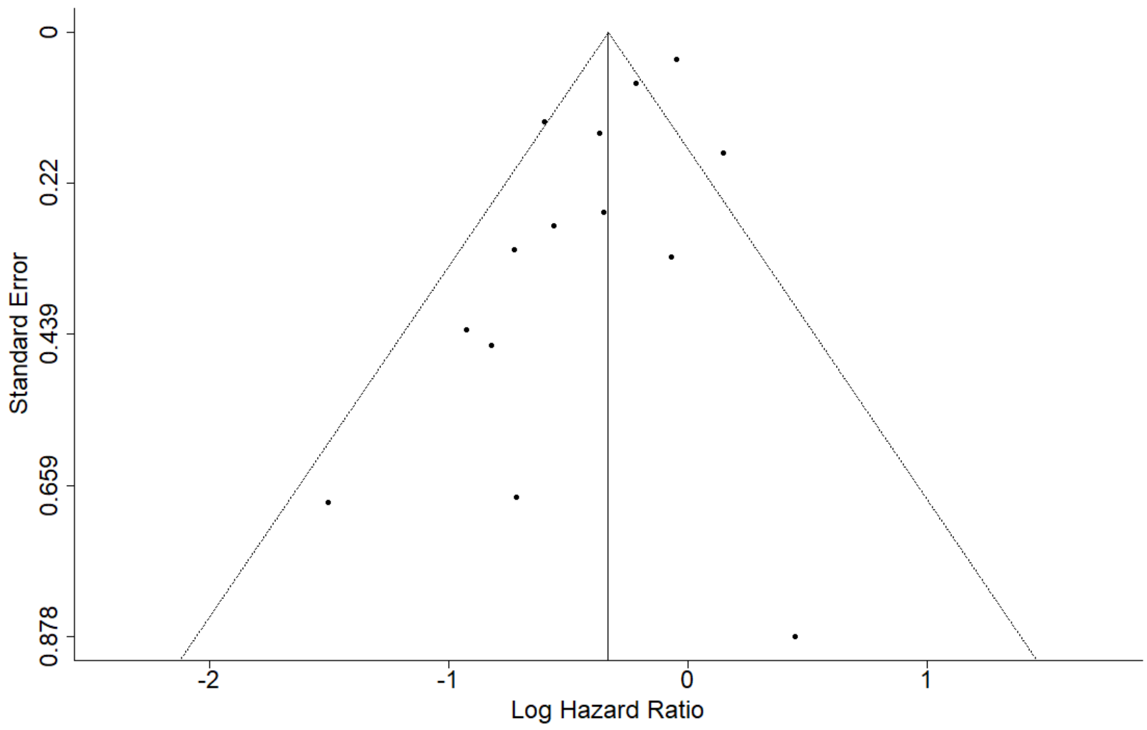

© 2019 Boyne DJ et al. *JAMA Network Open*.

eTable 1. Raw Data Extracted From the Studies Included in the Meta-analysis (n = 22)

Study	Chemotherapy Regime	Chemotherapy Duration	Overall Survival	Disease-Free Survival
**Randomized Clinical Trials**				
IDEA (2018)[9]	FOLFOX/CAPOX	6 months	-	Ref.
		3 months	**-**	HR: 1.05 (95% CI: 0.97-1.13)
Ito (2000)[10]	Carmofur	12 months	n = 50<br>5-year OS = 87%	n = 50<br>5-year DFS = 77%
		3 months	n = 94<br>5-year OS = 72%<br>g-Wilcoxon test = 0.0361<br>total events = 29	n = 94<br>5-year DFS = 63%<br>g-Wilcoxon test = 0.0487<br>total events = 39
**Observational Studies**				
Ahmed (2010)[11]	5-flourouracil	6 months	HR: 0.49 (95% CI: 0.38-0.64)	HR: 0.55 (95% CI: 0.42-0.70)
		1-5 months	Ref.	Ref.
Cespedes Feliciano (2017)[3]	FOLFOX	6-12 cycles	n = 491<br>OS higher in 6-12 month arm	Cespedes Feliciano (2017)
		1-5 cycles	n = 42<br>log-rank p-value = 0.04<br>Total events = 51	
Chapuis (2009)[4]	5-flourouracil	6 months	n = 68<br>3-year OS = 82%	-
		1-5 months	n = 36<br>3-year OS =72%<br>log-rank p-value = 0.030<br>total events = 40	-
Figer (2011)[5]	5-flourouracil	7-12 months	n = 305<br>greater OS relative to<br>1-6 month arm	-
		1-6 months	n = 93<br>log=rank p-value = 0.002<br>total events = 156	-

© 2019 Boyne DJ et al. *JAMA Network Open*.

Hassan (2015)[12]	5-flourouracil / capecitabine / FOLFOX / CAPOX	6 months	HR: 0.2 (95% CI: 0.06-0.64)	-
		1-5 months	Ref.	-
Hwang (2017)[13]	5-flourouracil / capecitabine	4-6 months	Ref.	-
		1-3 months	HR: 3.72 (95% CI: 2.80-4.94)	-
	FOLFOX / CAPOX	4-6 months	Ref.	-
		1-3 months	HR: 2.15 (95% CI: 1.87-2.47)	-
	tegafur	4-6 months	Ref.	-
		1-3 months	HR: 1.74 (95% CI: 0.56-5.41)	-
Ji (2018)[14]	FOLFOX	6 months	Ref.	Ref.
		4 months	HR: 0.958 (95% CI: 0.229-4.044)	HR: 0.743 (95% CI: 0.236-2.344)
Kim (2014)[6]	5-flourouracil / capecitabine / FOLFOX / CAPOX	75%+ cycles	n = 210<br>5-year OS = 80%	-
		< 75% cycles	n = 58<br>5-year OS = 64%<br>log-rank p-value = 0.0054<br>total events = 57	-
Kornmann (2008)[15]	5-flourouracil	14-39 weeks	HR: 0.4 (95% CI: 0.2-0.6)	-
		1-13 weeks	Ref.	-
Kumar (2015)[16]	FOLFOX	10-12 cycles	HR: 1.07 (95% CI: 0.70-1.61)	HR: 1.16 (95% CI: 0.82-1.63)
		1-9 cycles	Ref.	Ref.
Laurent (2018)[17]	FOLFOX	12 cycles	Ref.	-
		1-11 cycles	HR: 3.1 (95% CI: 1.1-8.5)	-
Morris (2007)[2]	5-flourouracil	4-6 cycles	HR: 0.55 (0.45-0.67)<br>vs. no chemotherapy	-
		1-3 cycles	HR: 1.09 (0.88 – 1.35)<br>vs. no chemotherapy	-
Neugut (2006)[18]	5-flourouracil	5-7 months	HR: 0.59 (0.49-0.71)	-
		1-4 months	Ref.	-
Qiu (2009)[19]	FOLFOX/ CAPOX/Capecitabine/5-flourouracil/tegafur	Slope	-	beta for ln(HR) per two month increase in chemotherapy duration = -0.618; se = 0.288

© 2019 Boyne DJ et al. *JAMA Network Open*.

Sgouros (2015)[7,a]	5-flourouracil / FOLFOX	6 month (stage II)	n = 61<br>3-year OS = 92.7	n = 61<br>3-year DFS = 96.3
		1-5 months (stage II)	n = 29<br>3-year OS = 88.2<br>log-rank p-value = 0.2179<br>total events = 15	n = 29<br>3-year DFS = 81.6<br>log-rank p-value = 0.0166<br>total events = 10
		6 month (stage III)	n = 78<br>3-year OS = 75.5	n = 78<br>3-year DFS = 61.9
		1-5 months (stage III)	n = 31<br>3-year OS = 83.2<br>log-rank p-value = 0.7313<br>total events = 42	n = 31<br>3-year DFS = 58.7<br>log-rank p-value = 0.6606<br>total events = 46
Sun (2015)[20]	Capecitabine	8 cycles	Ref.	Ref.
		1-7 cycles	HR: 3.27 (95% CI:1.44-7.45)	HR: 2.27 (0.93-5.53)
Tsai (2013)[21]	5-fluorouracil / tegafur	5-12 months	HR: 0.55 (95% CI: 0.42-0.73)	HR: 0.65 (95% CI: 0.49-0.87)
		1-4 months	Ref.	Ref.
Tsai (2016)[22]	FOLFOX	8-12 cycles	HR: 0.52 (95% CI: 0.28-0.97)	-
		1-7 cycles	Ref.	-
		7-12 cycles	-	HR: 0.57 (95% CI: 0.33-0.996)
		1-6 cycles	-	Ref.
van Erning (2017)[23]	Capecitabine	6-8 cycles	Ref.	Ref.
		1-5 cycles	HR: 2.00 (95% CI: 1.12-3.59)	HR: 2.07 (95% CI: 1.11-3.84)
	CAPOX	6-8 cycles	Ref.	Ref.
		1-5 cycles	HR: 1.17 (95% CI: 0.70-1.97)	HR: 1.42 (95% CI: 0.85-2.37)
Yun (2010)[8]	Capecitabine	8 cycles	-	n = 155;<br>3-year DFS = 90.7%
		1-7 cycles	-	n = 18;<br>3-year DFS = 70.9%;<br>log-rank test p-value = 0.028;<br>total events = 23

[a] The log-rank p-value and the total number of events were not reported in the original manuscript but were obtained directly from the authors
Abbreviation: HR = Hazard Ratio; OS = Overall Survival; DFS = Disease-free survival

© 2019 Boyne DJ et al. *JAMA Network Open*.

eTable 2. Reason for Risk of Bias Assignment in the Confounding and Selection Into the Study Domains

Study	Reason for assigning a Serious Risk of Bias due to Confounding	Moderate or Serious Risk of Bias due to Selection into the Study
Ahmed (2010)[11]	-	Serious risk of bias: the authors excluded patients lost to follow-up as well as "…patients who had inconclusive information about the number of treatment cycles…" and did not provide the number of patients excluded for these reasons. In addition, survival time was estimated starting from the time of surgery as opposed to the time of chemotherapy initiation.
Cespedes Feliciano (2017)[3]	No adjustment for age, sex, or stage	Moderate risk of bias: survival time was estimated starting from the time of diagnosis as opposed to the time of chemotherapy initiation.
Chapuis (2009)[4]	No adjustment for age or sex	Moderate risk of bias: survival time was estimated from the time of chemotherapy completion rather than the time of chemotherapy initiation.
Figer (2011)[5]	No adjustment for age, sex, stage, or site	Moderate risk of bias: survival time was estimated starting from the time of surgery as opposed to the time of chemotherapy initiation.
Hassan (2015)[12]	No adjustment for stage or chemotherapy regimen	Moderate risk of bias: survival time was estimated starting from the time of surgery as opposed to the time of chemotherapy initiation
Ji (2018)[14]	No adjustment for age, sex, or stage	-
Kim (2014)[6]	No adjustment for age or sex, or chemotherapy regimen	-
Morris (2007)[2]	-	Moderate risk of bias: survival time was estimated starting from the time of diagnosis as opposed to the time of chemotherapy initiation.
Neugut (2006)[18]	-	Serious risk of bias: excluded participants who, "were enrolled in a health maintenance organization (HMO) at any point from 12 months before to 8 months after initiation of FU chemotherapy… and/or were not covered by Medicare Parts A and B at any point during that period." Also excluded patients, "… who received

© 2019 Boyne DJ et al. *JAMA Network Open*.

		more than 7 months of treatment because they could not be distinguished from patients intended to receive longer (e.g., 12 month) regimens." In addition, survival time was also estimated from the time of diagnosis as opposed to the time of chemotherapy initiation.
Sgouros (2015)[7]	No adjustment for age, sex, site, or chemotherapy regimen	-
Sun (2015)[20]	No adjustment for age, sex, or stage	Moderate risk of bias: They excluded patients who had, "… less than 3 cycles of therapy for reasons other than toxicity…". In addition, survival time was also estimated from the time of diagnosis as opposed to the time of chemotherapy initiation.
Tsai (2016)[22]	-	Serious risk of bias: The authors excluded patients lost to follow-up without providing the number excluded. In addition, survival time was estimated starting from the time of surgery as opposed to the time of chemotherapy initiation.
Yun (2010)[8]	No adjustment age, sex, or stage	Moderate risk of bias: Survival time was estimated from the time of surgery as opposed to the time of chemotherapy initiation.

© 2019 Boyne DJ et al. *JAMA Network Open*.

References

1. Tierney JF, Stewart LA, Ghersi D, Burdett S, Sydes MR. Practical methods for incorporating summary time-to-event data into meta-analysis. *Trials.* 2007;8:16.
2. Morris M, Platell C, Fritschi L, Iacopetta B. Failure to complete adjuvant chemotherapy is associated with adverse survival in stage III colon cancer patients. *British journal of cancer.* 2007;96(5):701-707.
3. Cespedes Feliciano EM, Lee VS, Prado CM, et al. Muscle mass at the time of diagnosis of nonmetastatic colon cancer and early discontinuation of chemotherapy, delays, and dose reductions on adjuvant FOLFOX: The C-SCANS study. *Cancer.* 2017;123(24):4868-4877.
4. Chapuis PH, Bokey EL, Clarke S, Beale P, Dent OF. Adjuvant chemotherapy for stage C colonic cancer in a multidisciplinary setting. *ANZ journal of surgery.* 2009;79(10):685-692.
5. Figer A, Nissan A, Shani A, et al. Mature Results of a Prospective Randomized Trial Comparing 5-Flourouracil with Leucovorin to 5-Flourouracil with Levamisole as Adjuvant Therapy of Stage II and III Colorectal Cancer- The Israel Cooperative Oncology Group (ICOG) Study. *Journal of Cancer.* 2011;2:177-185.
6. Kim CA, Spratlin JL, Armstrong DE, Ghosh S, Mulder KE. Efficacy and safety of single agent or combination adjuvant chemotherapy in elderly patients with colon cancer: a Canadian cancer institute experience. *Clinical colorectal cancer.* 2014;13(3):199-206.
7. Sgouros J, Aravantinos G, Kouvatseas G, et al. Impact of Dose Reductions, Delays Between Chemotherapy Cycles, and/or Shorter Courses of Adjuvant Chemotherapy in Stage II and III Colorectal Cancer Patients: a Single-Center Retrospective Study. *Journal of gastrointestinal cancer.* 2015;46(4):343-349.
8. Yun JA, Kim HC, Son HS, et al. Oncologic outcome after cessation or dose reduction of capecitabine in patients with colon cancer. *Journal of the Korean Society of Coloproctology.* 2010;26(4):287-292.
9. Grothey A, Sobrero AF, Shields AF, et al. Duration of Adjuvant Chemotherapy for Stage III Colon Cancer. *The New England journal of medicine.* 2018;378(13):1177-1188.
10. Ito K, Kato T, Koike A, et al. Optimum duration of oral adjuvant chemotherapy of HCFU for colorectal cancer; review of 5-year follow-up. *Anticancer research.* 2000;20(6c):4681-4686.
11. Ahmed S, Ahmad I, Zhu T, et al. Early discontinuation but not the timing of adjuvant therapy affects survival of patients with high-risk colorectal cancer: a population-based study. *Diseases of the colon and rectum.* 2010;53(10):1432-1438.
12. Hassan AS, Naicker M, Yusof KH, Wan Ishak WZ. Prognostic factors and the role of adjuvant chemotherapy in post-curative surgery for Dukes B and C colon cancers and survival outcomes: a Malaysian experience. *Asian Pacific journal of cancer prevention : APJCP.* 2015;16(6):2237-2243.
13. Hwang IG, Lee JS, Lee S-C, Baek SK, Kim JG, Kim TW. Association between timing and duration of adjuvant chemotherapy and survival for colorectal cancer in korea, 2011-2014: A nationwide study based on the database of quality assessment and the health insurance. *Journal of Clinical Oncology.* 2017;35(15_suppl):3605-3605.
14. Ji WB, Hong KD, Kim JS, Joung SY, Um JW, Min BW. Effect of a Shortened Duration of FOLFOX Chemotherapy on the Survival Rate of Patients with Stage II and III Colon Cancer. *Chemotherapy.* 2018;63(1):8-12.
15. Kornmann M, Formentini A, Ette C, et al. Prognostic factors influencing the survival of patients with colon cancer receiving adjuvant 5-FU treatment. *European journal of surgical oncology : the journal of the European Society of Surgical Oncology and the British Association of Surgical Oncology.* 2008;34(12):1316-1321.

© 2019 Boyne DJ et al. *JAMA Network Open*.

16. Kumar A, Peixoto RD, Kennecke HF, et al. Effect of Adjuvant FOLFOX Chemotherapy Duration on Outcomes of Patients With Stage III Colon Cancer. *Clinical colorectal cancer.* 2015;14(4):262-268.e261.
17. Laurent M, Des Guetz G, Bastuji-Garin S, et al. Chronological Age and Risk of Chemotherapy Nonfeasibility: A Real-Life Cohort Study of 153 Stage II or III Colorectal Cancer Patients Given Adjuvant-modified FOLFOX6. *American journal of clinical oncology.* 2018;41(1):73-80.
18. Neugut AI, Matasar M, Wang X, et al. Duration of adjuvant chemotherapy for colon cancer and survival among the elderly. *Journal of clinical oncology : official journal of the American Society of Clinical Oncology.* 2006;24(15):2368-2375.
19. Qiu MZ, Teng KY, Ruan DY, et al. [Impact of adjuvant chemotherapy duration on 3-year disease-free survival of colorectal carcinoma patients after radical resection]. *Ai zheng = Aizheng = Chinese journal of cancer.* 2009;28(7):743-748.
20. Sun J, Ilich AI, Kim CA, et al. Concomitant Administration of Proton Pump Inhibitors and Capecitabine is Associated With Increased Recurrence Risk in Early Stage Colorectal Cancer Patients. *Clinical colorectal cancer.* 2016;15(3):257-263.
21. Tsai WS, Hsieh PS, Yeh CY, et al. Impact of chemotherapy-related prognostic factors on long-term survival in patients with stage III colorectal cancer after curative resection. *International journal of clinical oncology.* 2013;18(2):242-253.
22. Tsai YJ, Lin JK, Chen WS, et al. Adjuvant FOLFOX treatment for stage III colon cancer: how many cycles are enough? *SpringerPlus.* 2016;5(1):1318.
23. van Erning FN, Janssen-Heijnen ML, Creemers GJ, Pruijt JF, Maas HA, Lemmens VE. Recurrence-free and overall survival among elderly stage III colon cancer patients treated with CAPOX or capecitabine monotherapy. *International journal of cancer.* 2017;140(1):224-233.

© 2019 Boyne DJ et al. *JAMA Network Open*.
